# Posterior Shoulder Instability and Glenoid Bone Loss: A Review and a Free Bone Graft Technique

**DOI:** 10.3390/jcm13072016

**Published:** 2024-03-30

**Authors:** Walter Ryan Smith, T. Bradley Edwards

**Affiliations:** Fondren Orthopedic Group, Fondren Orthopedic Research Institute, Texas Orthopedic Hospital, Houston, TX 77030, USA; smithwalt1@gmail.com

**Keywords:** posterior shoulder instability, glenoid bone loss, iliac crest bone graft

## Abstract

Posterior glenoid bone loss (pGBL) is frequently associated with posterior shoulder instability. Posterior glenohumeral instability accounts for a small percentage of shoulder pathologies, and critical bone loss in posterior instability has not been well defined in the literature. Younger patient populations who participate in activities that repetitively stress the posterior stabilizing structures of the shoulder are more prone to developing posterior shoulder instability. A variety of surgical options have been described, ranging from isolated capsulolabral repair to glenoid osteotomy. Soft-tissue repair alone may be an inadequate treatment in cases of pGBL and places patients at a high risk of recurrence. Our preferred technique for posterior glenoid reconstruction in cases of pGBL involves the transfer of a free iliac crest bone graft onto the native glenoid. The graft is contoured to fit the osseous defect and secured to provide an extension of the glenoid track. In this study, we review pGBL in the setting of posterior instability and describe our technique in detail. Further long-term studies are needed to refine the indications for glenoid bone graft procedures and quantify what constitutes a critical pGBL.

## 1. Introduction

Posterior glenohumeral instability occurs from the disruption of the static and dynamic stabilizing structures of the shoulder. Traumatic posterior subluxation typically occurs with the arm in a flexed and internally rotated position, which places increased tension on the posterior–inferior glenohumeral ligament (PIGHL) and posterior labrum [1]. While significantly less common than anterior instability, posterior glenohumeral pathology may occur more frequently in certain individuals such as American football linemen, bench pressors, and military populations [2]. In addition to the soft-tissue stabilizers that act to balance the shoulder, the osseous anatomy of the shoulder, play a critical role in maintaining glenohumeral stability. Posterior glenoid bone loss (pGBL) is common in recurrent posterior instability and has been shown to be associated with increasing glenoid retroversion and glenoid hypoplasia [3,4]. After exhaustion of non-operative treatment, the mainstays of surgical treatment options include arthroscopic posterior capsulolabral repair [5,6], glenoid osteotomy [7], humeral rotational osteotomy [8], subscapularis transfer tenodesis (McLaughlin or modified McLaughlin procedure) [9], and glenoid bone graft procedures [10,11,12]. 

The focus of this paper is to review glenoid bone loss in the setting of posterior instability. We also describe our preferred surgical technique for glenoid reconstruction using a free bone graft from the iliac crest.

### 1.1. Initial Evaluation

As with any shoulder pathology, initial evaluation should include a comprehensive history, physical examination, and diagnostic imaging. As opposed to anterior instability, patients with posterior instability are less likely to report a frank dislocation or a traumatic event. Pain is most often times the presenting complaint [13]. However, in cases of substantial pGBL, patients are more likely to report symptoms of instability [3]. Posterior glenohumeral pathology may occur more frequently in certain individuals such as American football linemen, bench pressors, patients with seizure disorders, and military populations. These elements of a patient history should raise suspicion for posterior shoulder instability [1,2].

A thorough physical exam of the shoulder and cervical spine should be performed. The most notable provocative tests specific to posterior shoulder instability include the jerk test [14], the Kim test [15], and the posterior load and shift [16]. The jerk test is considered positive if the patient experiences a painful “jerk” as the examiner reduces the humeral head [14]. A positive Kim test and a positive posterior load and shift test are indicated by the reproduction of the patient’s pain or perceived feeling of instability [15]. Used in combination, these maneuvers have been shown to have high specificity and sensitivity for detecting posterior shoulder pathology [15]. In cases of substantial pGBL, the examiner may appreciate significant posterior translation of the humeral head, subluxation, or even complete dislocation of the patient’s glenohumeral joint.

### 1.2. Imaging of Posterior Glenoid Bone Loss

For patients with suspected shoulder instability, standard AP and rotational views of the shoulder should be obtained to evaluate associated pathologies such as a Hill–Sachs or reverse Hill–Sachs lesion [11]. Additionally, a glenoid profile view with the contralateral side for comparison should also be obtained to assess for posterior glenoid fracture or bone loss. CT or MRI arthrography may also prove helpful to further evaluate glenoid morphology and version, amount of bone erosion, glenoid fractures, and concomitant soft-tissue pathology. Neither of these modalities have been shown to be superior to one another with respect to the measurement of pGBL [17]. Three-dimensional CT reconstruction of the glenoid may be a useful adjunct in the evaluation of bone loss and morphology [17]. 

### 1.3. Quantifying the Critical pGBL

Patients with pGBL are at an increased risk for recurrent instability with soft-tissue repair alone [3]. However, the critical threshold of pGBL remains controversial. While several methods for the measurement of glenoid bone loss have been described, the most commonly used is the “perfect circle” method. Initially used in the context of anterior instability, this technique employs similar principles when used for the calculation of pGBL. Bone loss is calculated by dividing the width of the osseous defect by the diameter of a “perfect circle” placed on the inferior two-thirds of the glenoid [18]. It is prudent to point out that although anterior and posterior bone loss may be calculated in a similar fashion, the pathologic mechanisms differ significantly. Dekker et al. [19] found that posterior glenoid bone loss occurs at a mean of 30° off of the long axis of the glenoid. They concluded that posterior defects occur most frequently in the posteroinferior quadrant and extend on average from 6:44 to 9:28 on a clockface model. Critical bone loss in posterior instability from a quantitative standpoint has not been entirely defined. In a recent review, Dickens et al. [18] suggested that capsulolabral repair in isolation is inadequate in cases of pGBL >20% in the primary setting and >10% in revision cases. 

### 1.4. Surgical Treatment Options

Non-operative management may be attempted as an initial treatment strategy for posterior shoulder instability. Commonly utilized non-operative modalities include immobilization, activity modification or avoidance, anti-inflammatory medications, and physical therapy [1]. We define failure of non-operative treatment as persistent posterior instability or continued pain after at least six weeks of attempted non-surgical management. After failure of non-operative management, surgical intervention is frequently warranted for posterior shoulder instability, especially in the setting of pGBL. Arthroscopic capsulolabral repair can be considered in patients with preserved bone stock and isolated soft-tissue pathology [5]. Short-term outcomes have shown a high level of return to sport and patient satisfaction [20]. However, the long-term success rates after these soft-tissue procedures—lack of recurrent dislocation or subluxation—have been shown to be as low as 55% [3]. The outcomes are even less reliable if evaluated in the revision setting. Jewett et al. [21] found a high rate of persistent pain, recurrent instability, and dissatisfaction with clinical outcomes. Nacca et al. [22] showed in a biomechanical study that the loss of >20% of the posterior glenoid results in persistent instability after isolated capsulolabral repair. Additionally, Wolf et al. [3] noted a 44% failure with arthroscopic repair when pGBL was >13.5%, and Provencher et al. [23] found that a threshold of >11% was associated with a ten-time higher failure rate.

As mentioned previously, the relationship between glenoid retroversion and posterior instability is well established. Glenoid retroversion >10° has been identified as a risk factor for failure of soft-tissue repair in isolation [4]. In cases of excessive glenoid retroversion, an opening wedge glenoid osteotomy may be considered. Iliac crest autograft is typically used to fill the void left by the opening wedge. While this procedure is a powerful tool for restoring glenoid version, it is technically demanding surgery, and the complication rates have shown to be as high as 34% [24]. The recurrent instability rates are also not insignificant, as shown by an overall rate of 22% reported in a recent systematic review [25]. In a recent series of glenoid osteotomies by Waltenspül et al. [7], the authors found recurrent posterior shoulder instability in six of seven shoulders, as well as progression to osteoarthritis in 100% of the patients.

Less common procedures can be considered in certain scenarios. For example, a proximal humeral derotational osteotomy was described in the setting of a locked posterior dislocation of the shoulder. This salvage procedure may serve as a more appealing alternative to arthroplasty, especially in younger patients [26]. Surin et al. [8] found good or excellent results in 10 of 12 patients in their series at mid- to long-term follow-up. In cases of posterior instability with a large anterior humeral head defect, tenodesis of the supscapularis tendon or transfer of the lesser tuberosity into the defect can prevent the humeral head from engaging the posterior glenoid. This so-called McLaughlin procedure or other modified versions of the procedure may be performed as an open procedure or arthroscopically [9]. This technique can be performed in conjunction with other mentioned procedures that address posterior shoulder instability.

Various bone graft procedures to address posterior shoulder instability have been described. The rationale for this type of reconstruction is the extension of the glenoid track via transfer of a free bone graft to the posterior glenoid rim. In cases of pGBL or failed prior stabilization attempts, this procedure offers certain advantages (Table 1). Distinct advantages of this procedure include a low risk of recurrent dislocation, a high rate of return to sport, and potentially less morbidity than with a glenoid osteotomy [18]. Furthermore, because the primary stability of the procedure relies on native bone-to-bone healing (as opposed to soft-tissue or allograft healing), an accelerated return to activity may be possible [12]. While the indications for posterior augmentation are still being refined, bone graft augmentation in posterior instability may be considered in the setting of failed prior soft-tissue stabilization, poor-quality capsulolabral tissue, significant laxity, instability associated with glenoid dysplasia, large traumatic posterior Bankart lesions, and large reverse Hill–Sachs lesions [10]. It should be noted that the purpose of the bone graft is to serve as an extension of the glenoid track rather than as a mechanical “block”. Fried was the first surgeon to report a series of patients treated with posterior bone graft procedures in 1949 [27]. Recently, Mojica et al. [28] systematically reviewed 11 studies with combined 225 shoulders who underwent bone graft augmentation for posterior shoulder instability and found high patient-reported outcome scores and an overall recurrence rate of 9.8%. A number of posterior bone graft augmentation techniques have been described using either allograft or autograft. More recently, distal tibia allograft (DTA) has become a popular choice for reconstruction. The advantages of this procedure compared to autograft include decreased donor-site morbidity and similar congruity of the distal tibia articular surface to the glenoid rim. Arthroscopic and open techniques have been previously described [29]; however, long-term studies are lacking. The proposed disadvantages of allograft include a substantial cost and less biologic compatibility compared to autograft. DTA may also not be readily available in certain practice settings.

Multiple sources of autograft have been previously described for use in posterior shoulder reconstruction, including the iliac crest bone (iliac crest bone graft, ICBG) [11], the scapular spine, and the distal clavicle. While the scapular spine offers the advantage of obtaining the graft through the same incision as that for the reconstruction, this graft is typically less robust from a structural standpoint compared to ICBG. A bicortical ICBG is the authors’ bone graft of choice. Prior studies using an ICBG for posterior glenoid augmentation have demonstrated a low rate of pseudoarthrosis and graft resorption [30]. Mid-to-long-term studies demonstrated mixed clinical results. Servien et al. reported on 21 shoulders at a mean follow-up of 6 years. The majority of the patients returned to sport at their pre-injury level and had no evidence of glenohumeral arthritis at most recent follow-ups. They also found a low recurrence rate of posterior instability and high patient-reported outcome scores. In contrast, at a median of 18-year follow-up, Meuffels et al. [31] found a high rate of progression to osteoarthritis and inconsistent patient-reported outcomes after posterior bone-grafting procedures. However, as modern techniques have evolved, recent studies demonstrated more promising and consistently positive results. Camenzind et al. [32] reported on 19 shoulders treated with arthroscopic ICBG for recurrent posterior instability at a mean follow-up of 7.3 years. They found that 95% of the patients met or exceeded the satisfaction threshold of the Constant score. They also found statistically significant improvements in pain, ASES score, Rowe score, and subjective shoulder value. One potential explanation for improved outcome scores in more recent studies is a more precise placement of the graft on the glenoid rim. Specifically, newer techniques, such as our own, recommend contouring the ICBG to fit the glenoid defect. This minimizes the graft overhang and may potentially decrease undesirable contact forces between the humeral head and the bone graft that could lead to graft failure or the acceleration of osteoarthritis. Additionally, repairing the native capsule at the interface between the native glenoid and the bone block renders the graft extra-articular and provides a capsulorraphy effect. 

**Table 1:** Comparison of the advantages, disadvantages, and outcomes between open bone graft reconstruction of the glenoid and arthroscopic capsulolabral repair.

Procedure	Advantages	Disadvantages	Outcomes
Open Bone Graft Reconstruction	- Extension of glenoid track - Biologic healing of bone block to native glenoid - Ability to perform simultaneous capsulorraphy - Potentially accelerated return to sport or activity	- Donor site morbidity - Technically demanding - Risk of neurologic injury to suprascapular and axillary nerves - May require removal of symptomatic hardware	- Less than 10% risk of recurrent dislocation [28] - High Constant scores at mid-term follow-up [12] - Unclear risk of progression to osteoarthritis [31]
Arthroscopic Soft Tissue Repair	- Minimally invasive - Ability to address concurrent pathology in other areas of the shoulder - Improved scar cosmesis	- Unable to address glenoid bone loss and morphology - Higher recurrence rate in the long term	- High rate of return to sport [20] - High failure rate in the setting of pGBL [3,23]

Studies comparing allograft to autograft in posterior bone block procedures demonstrated both techniques as viable options. Nacca et al. [33] compared DTA and scapular spine autograft in a biomechanical study and found no significant differences in lateral displacement and peak forces. In a study evaluating DTA and ICBG, Frank et al. [34] found no differences in contact pressures when comparing both grafts to the intact glenoid. In other words, similar contact mechanics were found in the DTA and ICBG patient groups. Posterior glenoid reconstruction with a free bone graft can also be performed arthroscopically. Schwartz et al. [35] reported on a series of 19 shoulders with overall positive results, including radiologic bone healing in all cases. However, the authors emphasized a steep learning curve and the technically demanding nature of the procedure.

Loss of motion following stabilization procedures is a common concern. Gilat et al. [36] compared the pre-operative and the post-operative range of motion following posterior glenoid bone graft reconstruction in ten patients. They found no significant difference between the pre-operative and the post-operative range of motion at a minimum of a one-year follow-up. The authors also reported that only one patient reported recurrent instability after surgery. In a series of eight patients who underwent posterior glenoid reconstruction with ICBG, Barbier et al. [30] found that all patients recovered the normal range of motion in abduction and anterior elevation. They did note an external rotation deficit of 20° compared to the contralateral shoulder in three patients. These findings emphasize the importance of a precise graft placement, as loss of external rotation can occur if the bone graft is not placed flush with the native glenoid surface. The authors otherwise reported satisfactory results, with 80% of the patients demonstrating satisfactory results at a mean of a three-year follow-up. 

## 2. Surgical Technique

### 2.1. Indications

Posterior glenoid reconstruction with ICBG can be considered in patients with pGBL in either the primary or the revision setting. The authors consider pGBL > 15% with symptomatic instability to be the primary indication for free iliac crest bone graft transfer. Other strong indications for bone graft reconstruction include failed prior soft-tissue repair with recurrent instability, poor-quality or absent capsulolabral tissue, chronic glenoid fractures with persistent instability, glenoid dysplasia, and significant retroversion greater than 15° (Table 2). Additionally, posterior glenoid reconstruction with ICBG should be strongly considered in contact athletes, as these patients have a higher likelihood of recurrent instability than other patient populations. In cases of fixed posterior dislocations, these patients are often better served with a McLaughlin or modified McLaughlin procedure in order to address the significant reverse Hill–Sachs lesion. 

### 2.2. Pre-Operative Preparation and Positioning

General anesthesia with muscle paralysis should be utilized to aid during the joint exposure. We recommend the use of an interscalene block for post-operative pain control. The patient is placed in the lateral decubitus position with the non-operative side resting on the bed. This allows for the shoulder and iliac crest to be prepped into the same sterile field. An exam under anesthesia is performed prior to draping. The operative shoulder and pelvis are then prepped in standard sterile fashion from the anterior portion of the iliac crest to the superior aspect of the shoulder girdle. The operative arm is then placed across the body to aid during visualization.

### 2.3. Skin Incision, Initial Exposure, and Surgical Hazards

**Figure 1:**
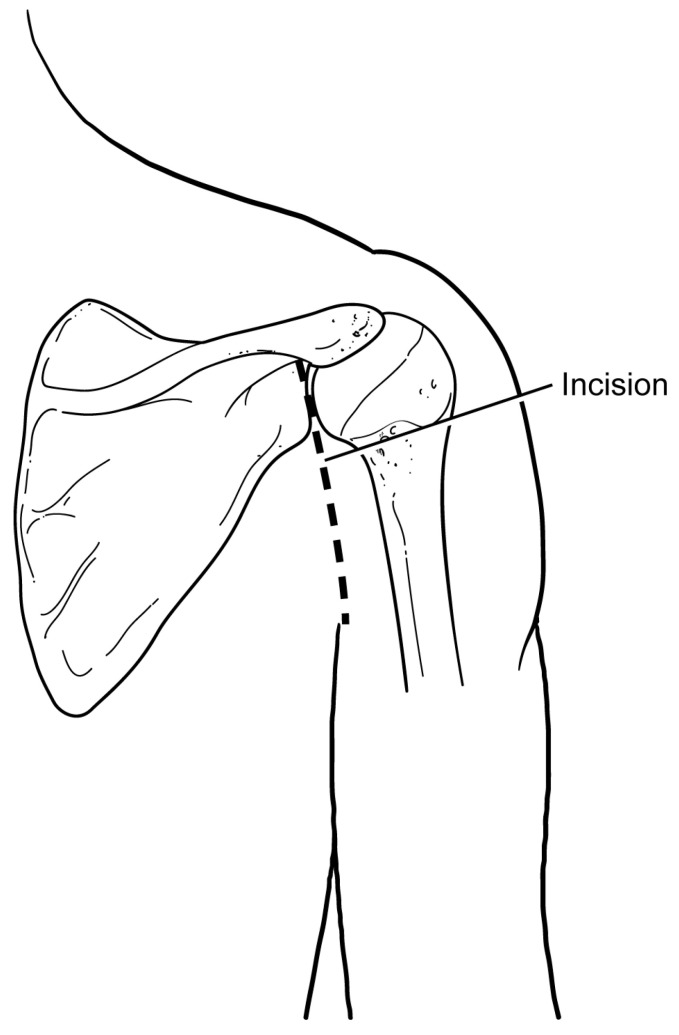
The surgical incision is made slightly medially to the posterolateral corner of the acromion. It is approximately 8–10 cm in length and extends distally towards the axillary crease.

**Figure 2:**
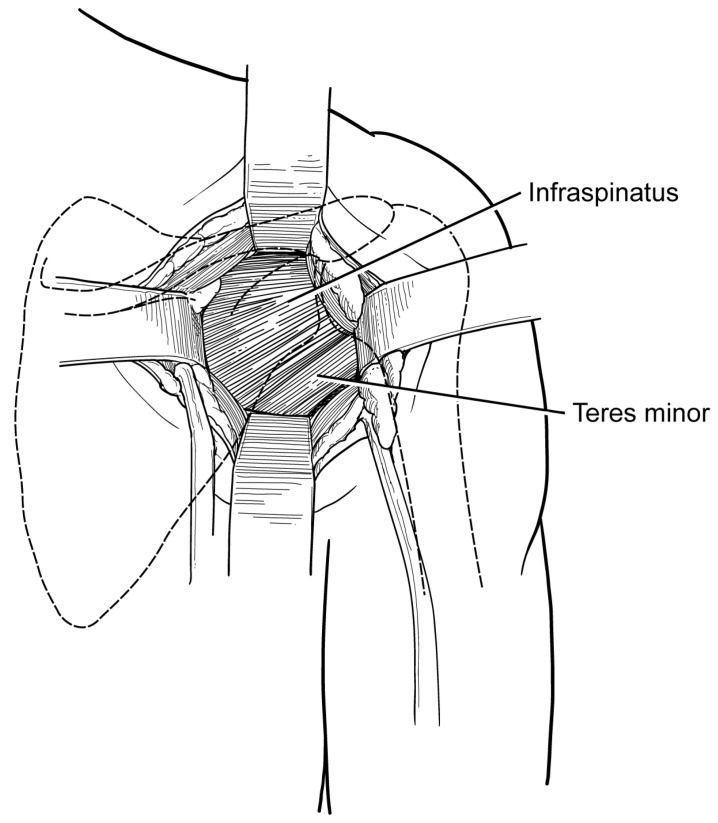
After the division of the skin and subcutaneous tissue, the deltoid is identified and retracted superiorly. The fascia overlying the infraspinatus muscle can then be visualized. The fascia is opened in line with the muscle fibers, which allows for access to the interval between the teres minor and the infraspinatus.

The authors prefer a standard posterior approach to the shoulder originally described by Brodsky et al. [37]. This approach to the shoulder is made utilizing an approximately 8 cm vertical incision originating just medial to the posterolateral tip of the acromion and extending distally towards the axilla (Figure 1). The deltoid fascia is identified and opened with an electrocautery device. The deltoid origin is protected and is not detached from the acromion. The inferior border of the deltoid is identified, and the muscle is retracted superiorly using a right-angle retractor. The fascia overlying the infraspinatus is then visualized and incised (Figure 2). The interval between the infraspinatus and the teres minor is then developed to allow for the visualization of the posterior glenohumeral joint capsule. Alternatively, the infraspinatus muscle may be split in line with its fibers in the lower portion. At this point, the humeral head and posterior glenoid can be easily palpated, and a vertical capsulotomy is made to expose the joint. Pointed Hohmann retractors are then placed on the superior and the inferior glenoid rim. 

The surgical hazards and anatomic relationships associated with the posterior approach to the shoulder are critical to understand in order to avoid neurovascular injury. Most notably, the suprascapular nerve passes adjacent to the base of the scapular spine and courses inferolaterally as it innervates the infraspinatus muscle. The nerve can be injured with a prolonged, aggressive retraction of the infraspinatus muscle. If the surgeon chooses to split the infraspinatus muscle, the suprascapular nerve can be palpated medially and protected. The axillary nerve and posterior circumflex humeral artery are also at risk during this approach. These structures course through the quadrangular space inferior to the teres minor muscle. Injury to these neurovascular structures can be avoided by correctly identifying the border of the teres minor and staying superior to the inferior border of the muscle.

### 2.4. Joint Exposure and Preparation

An intra-articular retractor is utilized to allow an assistant to retract the humeral head laterally. We prefer a smooth Trillat retractor. Alternatively, a Fukuda retractor may be used. The glenohumeral joint should now be well visualized, and lesions of the posterior labrum can be evaluated. The labral tissue may be preserved and laterally repaired. If there is a chronic fracture of the posterior glenoid, it can be excised at this point. A small curved osteotome may assist in removing any excess malunited bone from the posterior glenoid surface. Next, a burr is used to decorticate the posterior glenoid rim and form a smooth surface in preparation for receiving the bone block. 

### 2.5. Harvesting the Iliac Crest Bone Block

**Figure 3:**
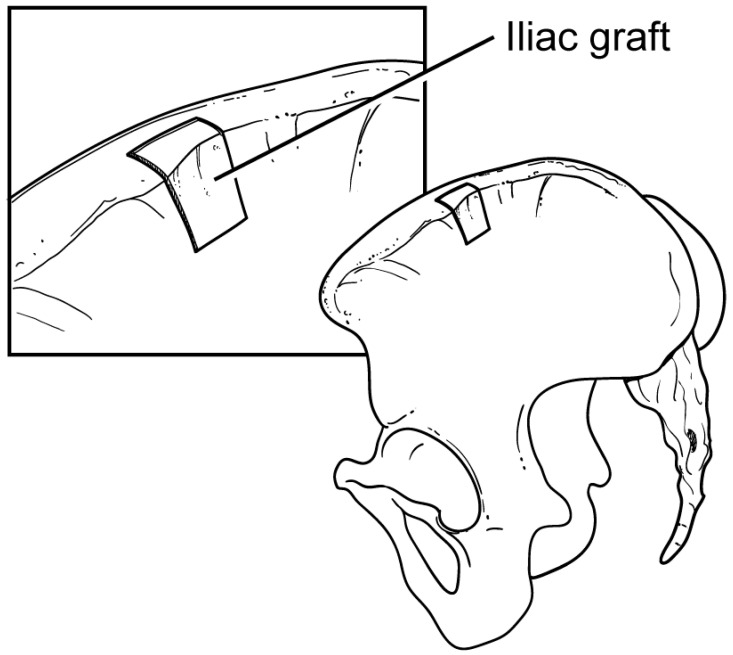
Prior to harvesting the bone block, the size of the glenoid defect is approximated so that the appropriate graft size can be taken. The bicortical iliac crest bone graft is taken from the superior and lateral cortices of the ilium, leaving the inner table intact.

Obtaining the graft may be performed simultaneously to the posterior approach to the shoulder if there is a qualified co-surgeon or assistant available. The incision to access the iliac crest is made starting 5 cm posteriorly to the anterior superior iliac spine extending in line with the pelvis towards the gluteus medius pillar. The oblique muscles and fascia are then divided and subperiosteally elevated using electrocautery to expose the inner and outer table of the pelvis. The dimensions of the bone block should generally match those of the defect. A ruler can be used to approximate the size of the defect before the graft is harvested. The graft is bicortical in nature; it is obtained from either the inner or the outer table and the superior cortex (Figure 3). A handheld sagittal saw is used to make the initial cuts to harvest the bone block, and curved osteotomes are used to complete the osteotomy. The bone block is then detached from the ilium, and the wound is packed until closure.

### 2.6. Preparation and Positioning of the Graft

**Figure 4:**
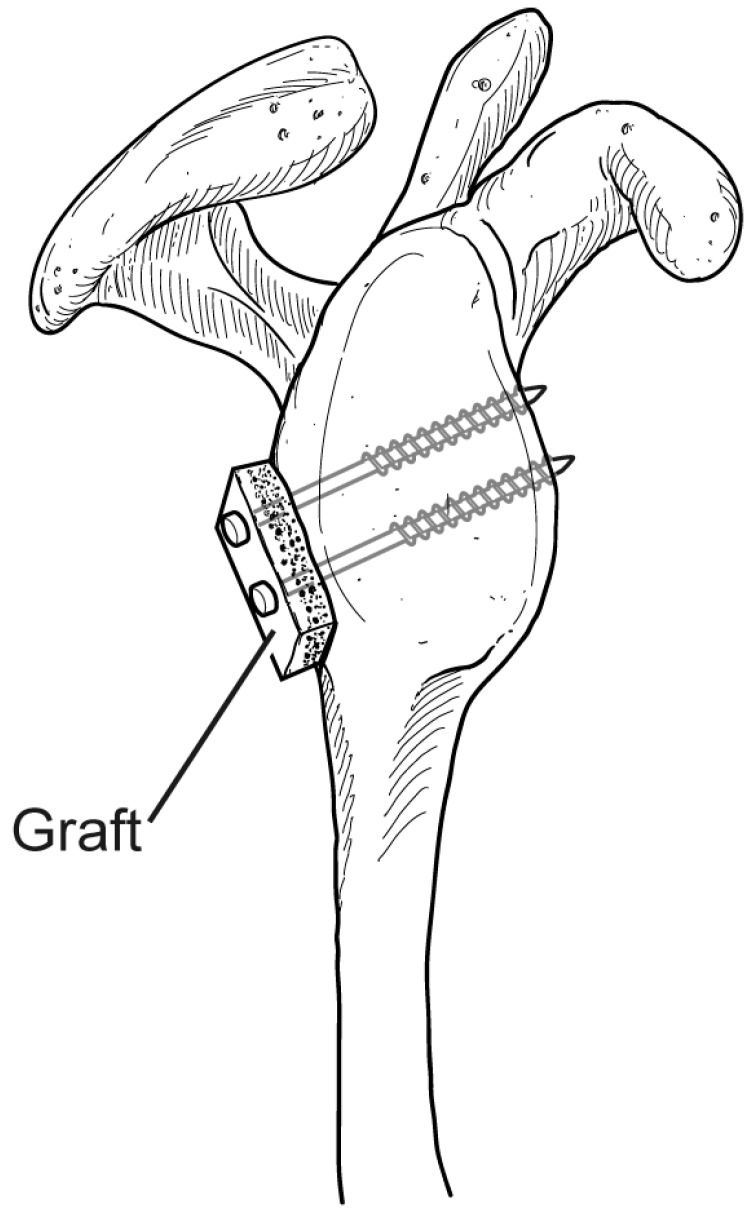
The bone graft is fixed to the glenoid rim using two 4.0 partially threaded cancellous screws. The screw trajectories should align perpendicularly to the graft–glenoid interface in order to achieve optimal compression and fixation.

The thickness of the cancellous portion of the graft should be preserved as much as possible. This aspect of the graft will ultimately face anteriorly and directly contact the native glenoid. The graft is contoured to fit the defect and is placed directly onto the posterior inferior glenoid rim. The bone block can be provisionally secured using guide wires for the cannulated screws and then inspected to ensure that it rests in the desired position. The graft should be placed essentially flush with the defect, with no lateral overhang. Once adequate positioning of the bone graft has been established, two partially threaded 4.0 cannulated screws (one inferior and one superior) with corresponding washers are placed to secure the graft to the native glenoid. The screw trajectories should be directed parallel to the articular surface of the glenoid (Figure 4). Once both screws have been placed, they should be sequentially tightened to ensure an adequate compression of the graft. Care should be taken not to overtighten the screws, as this can result in an iatrogenic fracture of the bone graft. If necessary, a burr can be used once more after the graft is secured for any additional contouring. 

### 2.7. Capsulolabral Repair and Closure

**Figure 5:**
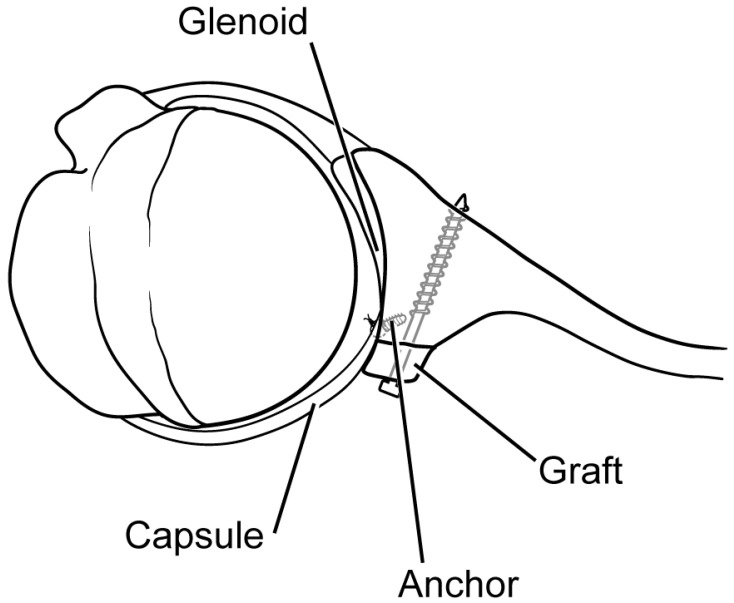
An axial image of the bone block is shown after it has been secured to the glenoid face. Capsular repair is performed at the interface between the glenoid and the bone graft, so as to render the graft extra-articular.

Capsulorraphy and labral repair (if possible) can then be performed after fixation of the graft has been achieved. Two anchors are placed (one superior and one inferior) into the native glenoid rim at the interface of the bone graft and the native articular surface. We prefer to use a 1.8 mm all-suture anchor. The sutures are then passed in a mattress fashion through the lateral aspect of the posterior capsule and labrum (if present) to complete the soft-tissue repair and make the graft extra-articular (Figure 5). The wound is then irrigated thoroughly and closed in a layered fashion.

### 2.8. Post-Operative Protocol

**Figure 6:**
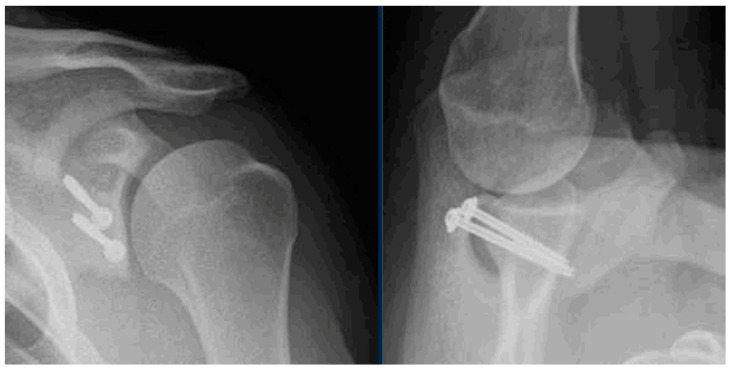
Grashey and axillary radiographs are obtained post-operatively. The XRs shown demonstrate appropriate position of the graft, flush with the native glenoid rim.

After surgery, the patient is placed in a simple sling for approximately two weeks. Post-operative radiographs are obtained at the first post-operative visit to ensure proper graft and hardware positioning (Figure 6). Physical therapy is initiated after the first post-operative visit, with a focus on gentle passive and active–assisted range-of-motion exercises. Internal rotation and adduction should be avoided to minimize the early loading of the bone graft. We prefer an aquatic therapy exercise regimen if possible. Active strengthening is initiated at twelve weeks, and return to sport is typically permitted at 4 to 6 months post-operatively. 

## Figures and Tables

**Table 2:** Authors’ preferred indications for posterior glenoid reconstruction with free iliac crest bone grafting.

Indications for Posterior Glenoid Reconstruction with ICBG
pGBL > 15% (in primary or revision setting)
Failed prior soft-tissue repair with recurrent instability
Poor-quality or deficient capsulolabral tissue
Chronic non-united posterior glenoid fractures
Glenoid dysplasia
Retroversion > 15°

## Data Availability

No new data were created or analyzed in this study. Data sharing is not applicable to this article.
